# Bis(diethyl­enetriamine)­cobalt(III) hexa­chloridoindate(III)

**DOI:** 10.1107/S1600536811016758

**Published:** 2011-05-07

**Authors:** Qihui Wu, Shangwen Chen, Cailing Zhang, Xia Zhi, Zelin Chen

**Affiliations:** a Department of Materials and Chemical Engineering, Ministry of Education Key Laboratory of Application Technology of Hainan, Superior Resources Chemical Materials, Hainan University, Haikou 570228, Hainan Province, People’s Republic of China

## Abstract

The title compound, [Co(C_4_H_13_N_3_)_2_][InCl_6_], was synthesized under hydro­thermal conditions. In the cation, the Co—N bond lengths lie in the range 1.967 (2)–1.9684 (15) Å. In the anion, the In^III^ atom is coordinated by six Cl atoms resulting in a slightly distorted octa­hedral geometry. Both metal atoms are located on special positions of site symmetry 2/*m*. Furthermore, one Cl atom and one N atom are located on a mirror plane. N—H⋯Cl hydrogen bonds between cations and anions consolidate the crystal packing.

## Related literature

For the use of chiral metal complexes as templates in the synthesis of open-framework metal phosphates and germanates, see: Stalder & Wilkinson (1997 ▶); Wang *et al.* (2003*a* ▶,*b* ▶); Pan *et al.* (2005 ▶, 2008 ▶). For the introduction of chiral metal complexes into coordination polymers, see: Pan *et al.* (2010*a* ▶,*b* ▶, 2011 ▶); Tong & Pan (2011 ▶). For In—Cl bond lengths in other hexa­chloridoindium compounds, see: Rothammel *et al.* (1998 ▶).
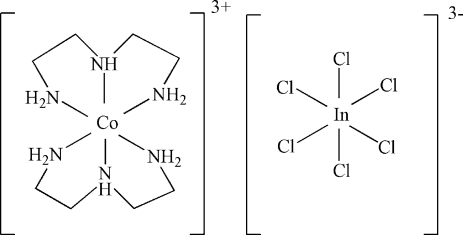


## Experimental

### 

#### Crystal data

[Co(C_4_H_13_N_3_)_2_][InCl_6_]
*M* *_r_* = 592.80
Orthorhombic, 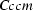

*a* = 10.8925 (5) Å
*b* = 14.7291 (7) Å
*c* = 12.2205 (6) Å
*V* = 1960.62 (16) Å^3^
*Z* = 4
Mo *K*α radiation
μ = 2.84 mm^−1^
*T* = 296 K
0.20 × 0.18 × 0.15 mm

#### Data collection

Bruker SMART APEX CCD area-detector diffractometer
Absorption correction: multi-scan (*SADABS*; Bruker, 2002 ▶) *T*_min_ = 0.572, *T*_max_ = 0.653
6910 measured reflections
1282 independent reflections
1139 reflections with *I* > 2σ(*I*)
*R*_int_ = 0.021

#### Refinement

*R*[*F* ^2^ > 2σ(*F* ^2^)] = 0.018
*wR*(*F* ^2^) = 0.046
*S* = 1.06
1282 reflections
57 parameters
H-atom parameters constrained
Δρ_max_ = 0.34 e Å^−3^
Δρ_min_ = −0.37 e Å^−3^

### 

Data collection: *APEX2* (Bruker, 2002 ▶); cell refinement: *SAINT* (Bruker, 2002 ▶); data reduction: *SAINT*; program(s) used to solve structure: *SHELXS97* (Sheldrick, 2008 ▶); program(s) used to refine structure: *SHELXL97* (Sheldrick, 2008 ▶); molecular graphics: *SHELXTL* (Sheldrick, 2008 ▶); software used to prepare material for publication: *SHELXTL*.

## Supplementary Material

Crystal structure: contains datablocks I, global. DOI: 10.1107/S1600536811016758/vm2092sup1.cif

Structure factors: contains datablocks I. DOI: 10.1107/S1600536811016758/vm2092Isup2.hkl

Additional supplementary materials:  crystallographic information; 3D view; checkCIF report

## Figures and Tables

**Table 1:** Hydrogen-bond geometry (Å, °)

*D*—H⋯*A*	*D*—H	H⋯*A*	*D*⋯*A*	*D*—H⋯*A*
N1—H1*A*⋯Cl1^i^	0.90	2.62	3.4915 (17)	164
N1—H1*B*⋯Cl2^ii^	0.90	2.61	3.3823 (18)	144
N1—H1*B*⋯Cl1^iii^	0.90	2.72	3.3957 (17)	133
N2—H2⋯Cl1^iv^	0.91	2.79	3.5407 (19)	141
N2—H2⋯Cl1^v^	0.91	2.79	3.5407 (19)	141

Symmetry codes: (i) 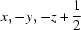; (ii) 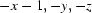; (iii) 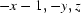; (iv) 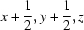; (v) 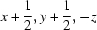.

**Fig. 1.**
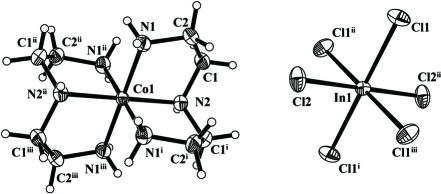
A view of the structure of complex. Ellipsoids are drawn at the 50% probability level.[Symmetry codes: (i) x,y,-z; (ii) -1/2 - x,-1/2 - y,z; (iii) -1/2 - x,-1/2 - y,-z.]

## supplementary crystallographic information

### Comment

Chiral metal complexes are interesting templates for the synthesis of novel materials, because they are versatile and can be made with a wide of shapes, charges and particularly chirality. As templates, they have been used in the synthesis of open-framework metal phosphates and germanates, (for example: Stalder & Wilkinson, 1997; Wang *et al.*, 2003*a*; Pan *et al.*, 2005, 2008) and a new concept of chirality transfer of the chiral metal complex into the inorganic framework has been demonstrated (Wang *et al.,* 2003*b*). Recently, Pan *et al.* introduced the chiral metal complexes into coordination polymers (Pan *et al.,* 2010*a*, 2010*b*, 2011; Tong & Pan, 2011).

In this paper, we present a new hexachloro-indium templated by the metal complex [Co(dien)_2_]^3+^. As shown in Fig. 1, the crystal structure consists of discrete [InCl_6_]^3-^ anions and [Co(dien)_2_]^3+^ cations. In [InCl_6_]^3-^, the indium center is coorinated by six Cl atoms, resulting in a slightly distorted octahedral geometry. The In—Cl bond distances are in the range of 2.5024 (5)–2.5114 (7) Å, which is consistent with other hexachloro-indium compounds (Rothammel *et al.*, 1998). In [Co(dien)_2_]^3+^, the cobalt center also displays a slightly distorted octahedral geometry and is bonded to six N atoms of two diethylenetriamines with the Co—N distances of 1.967 (2)–1.9684 (15) Å.

### Experimental

In a typical synthesis, a mixture of InCl_3_.4H_2_O (1 mmol), H_3_PO_4_ (4 mmol) Co(dien)_2_Cl_3_ (0.25 mmol) and H_2_O (10 ml) was added to a 20 ml Teflon-lined reactor under autogenous pressure at 100 °C for 3 days. Yellow block crystals were obtained.

### Refinement

All H atoms were positioned geometrically (C—H = 0.97 Å and N—H = 0.90-0.91 Å) and allowed to ride on their parent atoms, with *U*_iso_(H) = 1.2*U*_eq_(parent atom).

### Crystal data

**Table:**

[Co(C_4_H_13_N_3_)_2_][InCl_6_]	*F*(000) = 1176
*M**_r_* = 592.80	*D*_x_ = 2.008 Mg m^−^^3^
Orthorhombic, *C**c**c**m*	Mo *K*α radiation, λ = 0.71073 Å
Hall symbol: -C 2 2c	θ = 2.3–28.3°
*a* = 10.8925 (5) Å	µ = 2.84 mm^−^^1^
*b* = 14.7291 (7) Å	*T* = 296 K
*c* = 12.2205 (6) Å	Block, yellow
*V* = 1960.62 (16) Å^3^	0.2 × 0.18 × 0.15 mm
*Z* = 4	

### Data collection

**Table:**

Bruker SMART APEX CCD area-detector diffractometer	1282 independent reflections
Radiation source: fine-focus sealed tube	1139 reflections with *I* > 2σ(*I*)
graphite	*R*_int_ = 0.021
Detector resolution: 5.00cm pixels mm^-1^	θ_max_ = 28.3°, θ_min_ = 2.3°
φ and ω scans	*h* = −14→11
Absorption correction: multi-scan (*SADABS*; Bruker, 2002)	*k* = −19→18
*T*_min_ = 0.572, *T*_max_ = 0.653	*l* = −12→16
6910 measured reflections	

### Refinement

**Table:**

Refinement on *F*^2^	Primary atom site location: structure-invariant direct methods
Least-squares matrix: full	Secondary atom site location: difference Fourier map
*R*[*F*^2^ > 2σ(*F*^2^)] = 0.018	Hydrogen site location: inferred from neighbouring sites
*wR*(*F*^2^) = 0.046	H-atom parameters constrained
*S* = 1.06	*w* = 1/[σ^2^(*F*_o_^2^) + (0.0179*P*)^2^ + 3.2797*P*] where *P* = (*F*_o_^2^ + 2*F*_c_^2^)/3
1282 reflections	(Δ/σ)_max_ < 0.001
57 parameters	Δρ_max_ = 0.34 e Å^−^^3^
0 restraints	Δρ_min_ = −0.37 e Å^−^^3^

### Special details

**Table:**

Geometry. All e.s.d.'s (except the e.s.d. in the dihedral angle between two l.s. planes) are estimated using the full covariance matrix. The cell e.s.d.'s are taken into account individually in the estimation of e.s.d.'s in distances, angles and torsion angles; correlations between e.s.d.'s in cell parameters are only used when they are defined by crystal symmetry. An approximate (isotropic) treatment of cell e.s.d.'s is used for estimating e.s.d.'s involving l.s. planes.
Refinement. Refinement of *F*^2^ against ALL reflections. The weighted *R*-factor *wR* and goodness of fit *S* are based on *F*^2^, conventional *R*-factors *R* are based on *F*, with *F* set to zero for negative *F*^2^. The threshold expression of *F*^2^ > σ(*F*^2^) is used only for calculating *R*-factors(gt) *etc*. and is not relevant to the choice of reflections for refinement. *R*-factors based on *F*^2^ are statistically about twice as large as those based on *F*, and *R*- factors based on ALL data will be even larger.

### Fractional atomic coordinates and isotropic or equivalent isotropic displacement parameters (Å^2^)

**Table:**

	*x*	*y*	*z*	*U*_iso_*/*U*_eq_	
In1	−0.2500	−0.2500	0.0000	0.02018 (8)	
Co1	−0.2500	0.2500	0.0000	0.01769 (10)	
Cl1	−0.38483 (5)	−0.31981 (4)	0.14285 (4)	0.03547 (12)	
Cl2	−0.38713 (6)	−0.11293 (4)	0.0000	0.03569 (17)	
N1	−0.35382 (15)	0.19629 (10)	0.11486 (13)	0.0265 (3)	
H1A	−0.3447	0.2282	0.1772	0.080*	
H1B	−0.4331	0.1999	0.0945	0.080*	
N2	−0.1606 (2)	0.13392 (14)	0.0000	0.0228 (4)	
H2	−0.0789	0.1466	0.0000	0.080*	
C1	−0.18921 (19)	0.08352 (13)	0.10310 (16)	0.0289 (4)	
H1C	−0.1353	0.1041	0.1612	0.080*	
H1D	−0.1753	0.0191	0.0921	0.080*	
C2	−0.32142 (19)	0.09943 (13)	0.13544 (17)	0.0295 (4)	
H2A	−0.3748	0.0601	0.0931	0.080*	
H2B	−0.3326	0.0853	0.2123	0.080*	

### Atomic displacement parameters (Å^2^)

**Table:**

	*U*^11^	*U*^22^	*U*^33^	*U*^12^	*U*^13^	*U*^23^
In1	0.01848 (12)	0.02348 (13)	0.01860 (12)	0.00027 (9)	0.000	0.000
Co1	0.0176 (2)	0.0165 (2)	0.0190 (2)	−0.00096 (17)	0.000	0.000
Cl1	0.0303 (2)	0.0454 (3)	0.0307 (2)	−0.0003 (2)	0.0080 (2)	0.0119 (2)
Cl2	0.0245 (3)	0.0231 (3)	0.0595 (5)	0.0016 (2)	0.000	0.000
N1	0.0266 (8)	0.0242 (7)	0.0286 (8)	−0.0011 (6)	0.0058 (6)	0.0023 (6)
N2	0.0220 (10)	0.0210 (10)	0.0255 (11)	0.0000 (8)	0.000	0.000
C1	0.0342 (10)	0.0247 (9)	0.0277 (9)	0.0031 (8)	−0.0027 (8)	0.0049 (7)
C2	0.0352 (11)	0.0234 (9)	0.0298 (10)	−0.0029 (8)	0.0031 (8)	0.0056 (7)

### Geometric parameters (Å, °)

**Table:**

In1—Cl1^i^	2.5024 (5)	N1—C2	1.491 (2)
In1—Cl1^ii^	2.5024 (5)	N1—H1A	0.9000
In1—Cl1	2.5024 (5)	N1—H1B	0.9000
In1—Cl1^iii^	2.5024 (5)	N2—C1^i^	1.495 (2)
In1—Cl2^iii^	2.5114 (7)	N2—C1	1.495 (2)
In1—Cl2	2.5114 (7)	N2—H2	0.9100
Co1—N2	1.967 (2)	C1—C2	1.512 (3)
Co1—N2^iv^	1.967 (2)	C1—H1C	0.9700
Co1—N1^i^	1.9684 (15)	C1—H1D	0.9700
Co1—N1^v^	1.9684 (15)	C2—H2A	0.9700
Co1—N1^iv^	1.9684 (15)	C2—H2B	0.9700
Co1—N1	1.9684 (15)		
			
Cl1^i^—In1—Cl1^ii^	180.0	N1^i^—Co1—N1	90.97 (10)
Cl1^i^—In1—Cl1	88.48 (3)	N1^v^—Co1—N1	89.03 (10)
Cl1^ii^—In1—Cl1	91.52 (3)	N1^iv^—Co1—N1	180.00 (8)
Cl1^i^—In1—Cl1^iii^	91.52 (3)	C2—N1—Co1	111.65 (12)
Cl1^ii^—In1—Cl1^iii^	88.48 (3)	C2—N1—H1A	109.3
Cl1—In1—Cl1^iii^	180.0	Co1—N1—H1A	109.3
Cl1^i^—In1—Cl2^iii^	91.076 (16)	C2—N1—H1B	109.3
Cl1^ii^—In1—Cl2^iii^	88.924 (17)	Co1—N1—H1B	109.3
Cl1—In1—Cl2^iii^	91.076 (16)	H1A—N1—H1B	108.0
Cl1^iii^—In1—Cl2^iii^	88.924 (17)	C1^i^—N2—C1	114.8 (2)
Cl1^i^—In1—Cl2	88.924 (17)	C1^i^—N2—Co1	109.18 (12)
Cl1^ii^—In1—Cl2	91.076 (16)	C1—N2—Co1	109.18 (12)
Cl1—In1—Cl2	88.924 (17)	C1^i^—N2—H2	107.8
Cl1^iii^—In1—Cl2	91.076 (16)	C1—N2—H2	107.8
Cl2^iii^—In1—Cl2	180.0	Co1—N2—H2	107.8
N2—Co1—N2^iv^	180.0	N2—C1—C2	109.98 (16)
N2—Co1—N1^i^	86.27 (6)	N2—C1—H1C	109.7
N2^iv^—Co1—N1^i^	93.73 (6)	C2—C1—H1C	109.7
N2—Co1—N1^v^	93.73 (6)	N2—C1—H1D	109.7
N2^iv^—Co1—N1^v^	86.27 (6)	C2—C1—H1D	109.7
N1^i^—Co1—N1^v^	180.00 (10)	H1C—C1—H1D	108.2
N2—Co1—N1^iv^	93.73 (6)	N1—C2—C1	109.24 (15)
N2^iv^—Co1—N1^iv^	86.27 (6)	N1—C2—H2A	109.8
N1^i^—Co1—N1^iv^	89.03 (10)	C1—C2—H2A	109.8
N1^v^—Co1—N1^iv^	90.97 (10)	N1—C2—H2B	109.8
N2—Co1—N1	86.27 (6)	C1—C2—H2B	109.8
N2^iv^—Co1—N1	93.73 (6)	H2A—C2—H2B	108.3

Symmetry codes: (i) *x*, *y*, −*z*; (ii) −*x*−1/2, −*y*−1/2, *z*; (iii) −*x*−1/2, −*y*−1/2, −*z*; (iv) −*x*−1/2, −*y*+1/2, −*z*; (v) −*x*−1/2, −*y*+1/2, *z*.

### Hydrogen-bond geometry (Å, °)

**Table:**

*D*—H···*A*	*D*—H	H···*A*	*D*···*A*	*D*—H···*A*
N1—H1A···Cl1^vi^	0.90	2.62	3.4915 (17)	164
N1—H1B···Cl2^vii^	0.90	2.61	3.3823 (18)	144
N1—H1B···Cl1^viii^	0.90	2.72	3.3957 (17)	133
N2—H2···Cl1^ix^	0.91	2.79	3.5407 (19)	141
N2—H2···Cl1^x^	0.91	2.79	3.5407 (19)	141

Symmetry codes: (vi) *x*, −*y*, −*z*+1/2; (vii) −*x*−1, −*y*, −*z*; (viii) −*x*−1, −*y*, *z*; (ix) *x*+1/2, *y*+1/2, *z*; (x) *x*+1/2, *y*+1/2, −*z*.
